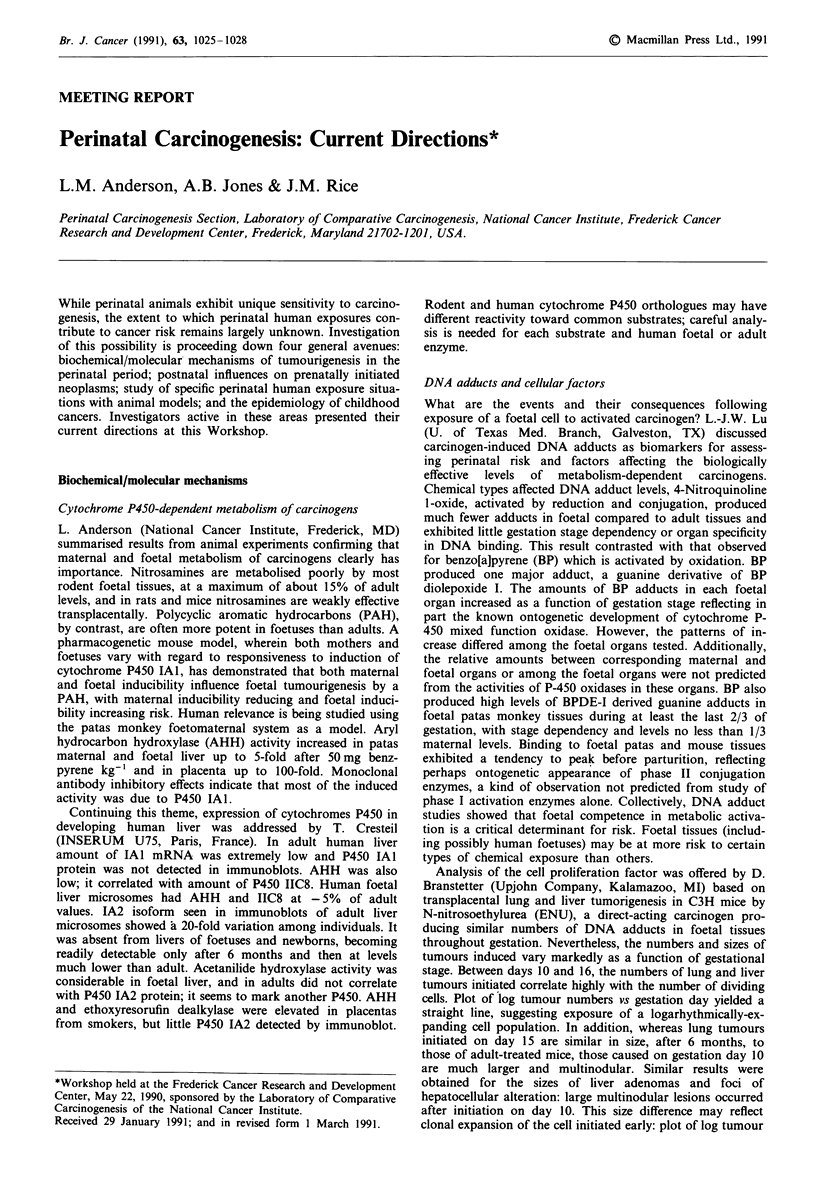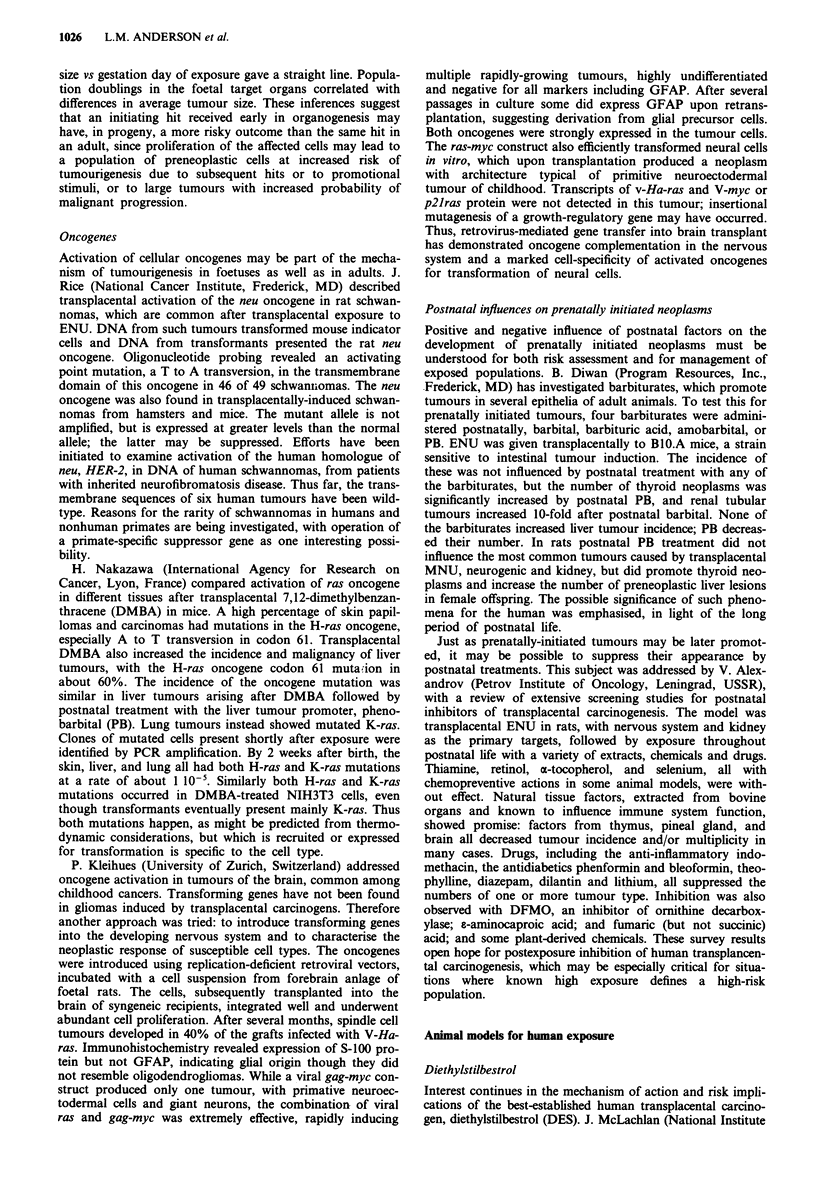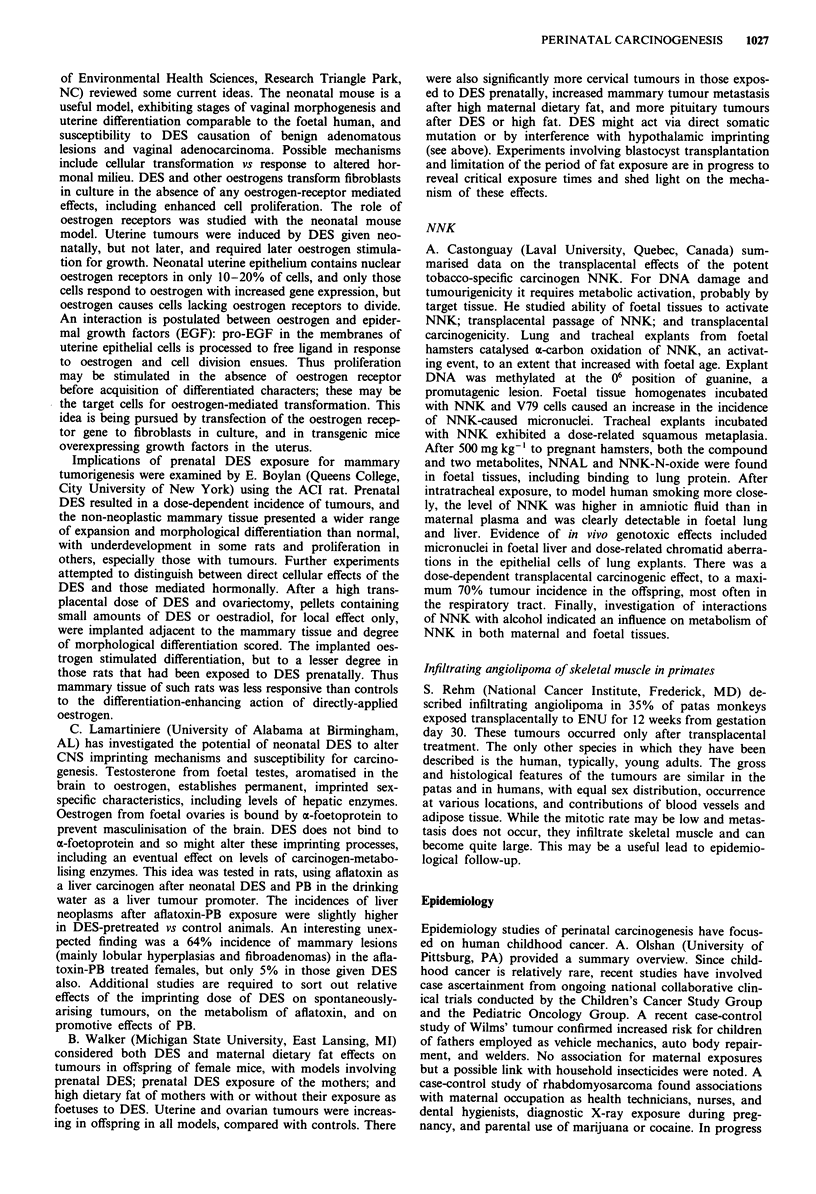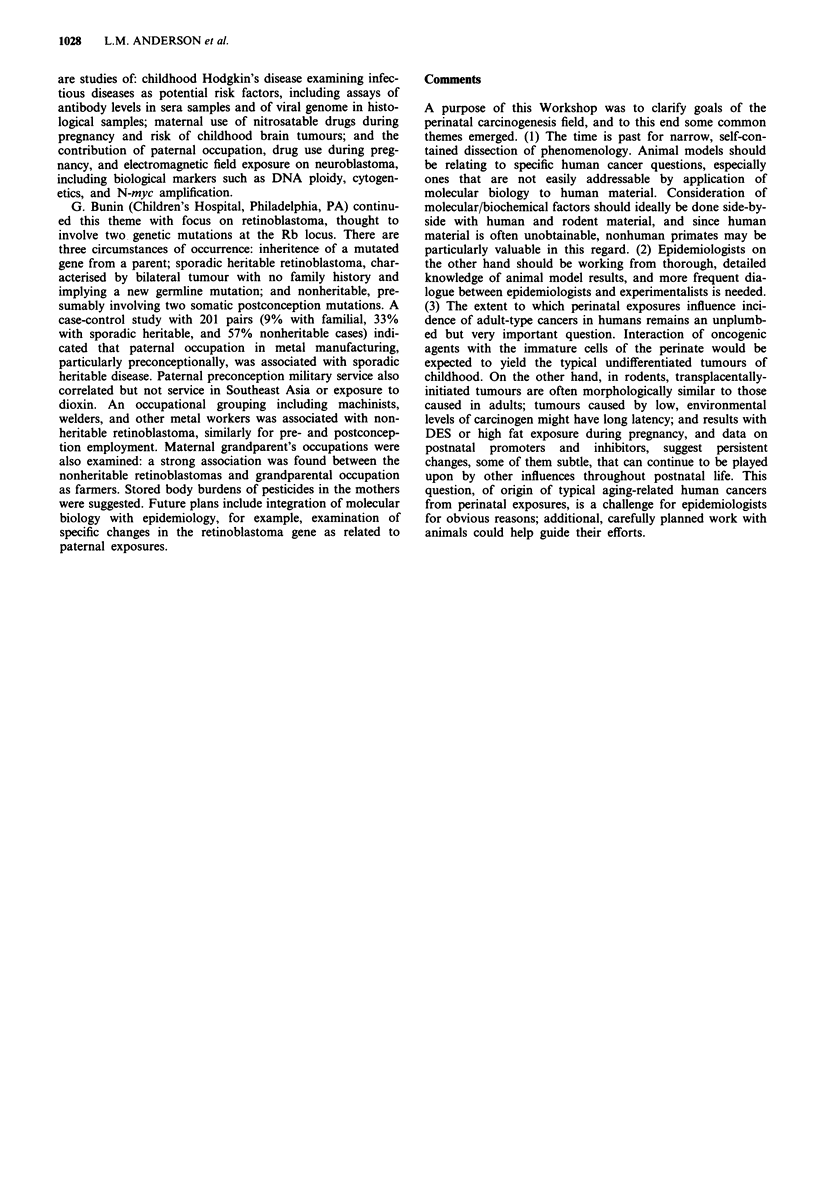# Perinatal carcinogenesis: current directions.

**DOI:** 10.1038/bjc.1991.224

**Published:** 1991-06

**Authors:** L. M. Anderson, A. B. Jones, J. M. Rice

**Affiliations:** Laboratory of Comparative Carcinogenesis, National Cancer Institute, Frederick Cancer Research and Development Center, Maryland 21702-1201.


Br. J. Cancer (1991), 63, 1025–1028 © Macmillan Press Ltd., 1991

MEETING REPORT

### Perinatal Carcinogenesis: Current Directions*

L.M. Anderson, A.B. Jones & J.M. Rice

*Perinatal Carcinogenesis Section, Laboratory of Comparative Carcinogenesis, National Cancer Institute, Frederick Cancer Research and Development Center, Frederick, Maryland 21702-1201, USA.*

While perinatal animals exhibit unique sensitivity to carcinogenesis, the extent to which perinatal human exposures contribute to cancer risk remains largely unknown. Investigation of this possibility is proceeding down four general avenues: biochemical/molecular mechanisms of tumourigenesis in the perinatal period; postnatal influences on prenatally initiated neoplasms; study of specific perinatal human exposure situations with animal models; and the epidemiology of childhood cancers. Investigators active in these areas presented their current directions at this Workshop.

### Biochemical/molecular mechanisms

### *Cytochrome P450-dependent metabolism of carcinogens*

L. Anderson (National Cancer Institute, Frederick, MD) summarised results from animal experiments confirming that maternal and foetal metabolism of carcinogens clearly has importance. Nitrosamines are metabolised poorly by most rodent foetal tissues, at a maximum of about 15% of adult levels, and in rats and mice nitrosamines are weakly effective transplacentally. Polycyclic aromatic hydrocarbons (PAH), by contrast, are often more potent in foetuses than adults. A pharmacogenetic mouse model, wherein both mothers and foetuses vary with regard to responsiveness to induction of cytochrome P450 IA1, has demonstrated that both maternal and foetal inducibility influence foetal tumourigenesis by a PAH, with maternal inducibility reducing and foetal inducibility increasing risk. Human relevance is being studied using the patas monkey foetomaternal system as a model. Aryl hydrocarbon hydroxylase (AHH) activity increased in patas maternal and foetal liver up to 5-fold after 50 mg benzpyrene $kg^{-1}$ and in placenta up to 100-fold. Monoclonal antibody inhibitory effects indicate that most of the induced activity was due to P450 IA1.

Continuing this theme, expression of cytochromes P450 in developing human liver was addressed by T. Cresteil (INSERUM U75, Paris, France). In adult human liver amount of IA1 mRNA was extremely low and P450 IA1 protein was not detected in immunoblots. AHH was also low; it correlated with amount of P450 IIC8. Human foetal liver microsomes had AHH and IIC8 at −5% of adult values. IA2 isoform seen in immunoblots of adult liver microsomes showed a 20-fold variation among individuals. It was absent from livers of foetuses and newborns, becoming readily detectable only after 6 months and then at levels much lower than adult. Acetanilide hydroxylase activity was considerable in foetal liver, and in adults did not correlate with P450 IA2 protein; it seems to mark another P450. AHH and ethoxyresorufin dealkylase were elevated in placentas from smokers, but little P450 IA2 detected by immunoblot. Rodent and human cytochrome P450 orthologues may have different reactivity toward common substrates; careful analysis is needed for each substrate and human foetal or adult enzyme.

### *DNA adducts and cellular factors*

What are the events and their consequences following exposure of a foetal cell to activated carcinogen? L.-J.W. Lu (U. of Texas Med. Branch, Galveston, TX) discussed carcinogen-induced DNA adducts as biomarkers for assessing perinatal risk and factors affecting the biologically effective levels of metabolism-dependent carcinogens. Chemical types affected DNA adduct levels, 4-Nitroquinoline 1-oxide, activated by reduction and conjugation, produced much fewer adducts in foetal compared to adult tissues and exhibited little gestation stage dependency or organ specificity in DNA binding. This result contrasted with that observed for benzo[a]pyrene (BP) which is activated by oxidation. BP produced one major adduct, a guanine derivative of BP diolepoxide I. The amounts of BP adducts in each foetal organ increased as a function of gestation stage reflecting in part the known ontogenetic development of cytochrome P-450 mixed function oxidase. However, the patterns of increase differed among the foetal organs tested. Additionally, the relative amounts between corresponding maternal and foetal organs or among the foetal organs were not predicted from the activities of P-450 oxidases in these organs. BP also produced high levels of BPDE-I derived guanine adducts in foetal patas monkey tissues during at least the last 2/3 of gestation, with stage dependency and levels no less than 1/3 maternal levels. Binding to foetal patas and mouse tissues exhibited a tendency to peak before parturition, reflecting perhaps ontogenetic appearance of phase II conjugation enzymes, a kind of observation not predicted from study of phase I activation enzymes alone. Collectively, DNA adduct studies showed that foetal competence in metabolic activation is a critical determinant for risk. Foetal tissues (including possibly human foetuses) may be at more risk to certain types of chemical exposure than others.

Analysis of the cell proliferation factor was offered by D. Branstetter (Upjohn Company, Kalamazoo, MI) based on transplacental lung and liver tumorigenesis in C3H mice by N-nitrosoethylurea (ENU), a direct-acting carcinogen producing similar numbers of DNA adducts in foetal tissues throughout gestation. Nevertheless, the numbers and sizes of tumours induced vary markedly as a function of gestational stage. Between days 10 and 16, the numbers of lung and liver tumours initiated correlate highly with the number of dividing cells. Plot of log tumour numbers *vs* gestation day yielded a straight line, suggesting exposure of a logarhythmically-expanding cell population. In addition, whereas lung tumours initiated on day 15 are similar in size, after 6 months, to those of adult-treated mice, those caused on gestation day 10 are much larger and multinodular. Similar results were obtained for the sizes of liver adenomas and foci of hepatocellular alteration: large multinodular lesions occurred after initiation on day 10. This size difference may reflect clonal expansion of the cell initiated early: plot of log tumour

*Workshop held at the Frederick Cancer Research and Development Center, May 22, 1990, sponsored by the Laboratory of Comparative Carcinogenesis of the National Cancer Institute.

Received 29 January 1991; and in revised form 1 March 1991.

size *vs* gestation day of exposure gave a straight line. Population doublings in the foetal target organs correlated with differences in average tumour size. These inferences suggest that an initiating hit received early in organogenesis may have, in progeny, a more risky outcome than the same hit in an adult, since proliferation of the affected cells may lead to a population of preneoplastic cells at increased risk of tumourigenesis due to subsequent hits or to promotional stimuli, or to large tumours with increased probability of malignant progression.

### *Oncogenes*

Activation of cellular oncogenes may be part of the mechanism of tumourigenesis in foetuses as well as in adults. J. Rice (National Cancer Institute, Frederick, MD) described transplacental activation of the *neu* oncogene in rat schwannomas, which are common after transplacental exposure to ENU. DNA from such tumours transformed mouse indicator cells and DNA from transformants presented the rat *neu* oncogene. Oligonucleotide probing revealed an activating point mutation, a T to A transversion, in the transmembrane domain of this oncogene in 46 of 49 schwannomas. The *neu* oncogene was also found in transplacentally-induced schwannomas from hamsters and mice. The mutant allele is not amplified, but is expressed at greater levels than the normal allele; the latter may be suppressed. Efforts have been initiated to examine activation of the human homologue of *neu*, *HER-2*, in DNA of human schwannomas, from patients with inherited neurofibromatosis disease. Thus far, the transmembrane sequences of six human tumours have been wild-type. Reasons for the rarity of schwannomas in humans and nonhuman primates are being investigated, with operation of a primate-specific suppressor gene as one interesting possibility.

H. Nakazawa (International Agency for Research on Cancer, Lyon, France) compared activation of *ras* oncogene in different tissues after transplacental 7,12-dimethylbenzanthracene (DMBA) in mice. A high percentage of skin papillomas and carcinomas had mutations in the H-*ras* oncogene, especially A to T transversion in codon 61. Transplacental DMBA also increased the incidence and malignancy of liver tumours, with the H-*ras* oncogene codon 61 mutation in about 60%. The incidence of the oncogene mutation was similar in liver tumours arising after DMBA followed by postnatal treatment with the liver tumour promoter, phenobarbital (PB). Lung tumours instead showed mutated K-*ras*. Clones of mutated cells present shortly after exposure were identified by PCR amplification. By 2 weeks after birth, the skin, liver, and lung all had both H-*ras* and K-*ras* mutations at a rate of about 1 $10^{-5}$. Similarly both H-*ras* and K-*ras* mutations occurred in DMBA-treated NIH3T3 cells, even though transformants eventually present mainly K-*ras*. Thus both mutations happen, as might be predicted from thermodynamic considerations, but which is recruited or expressed for transformation is specific to the cell type.

P. Kleihues (University of Zurich, Switzerland) addressed oncogene activation in tumours of the brain, common among childhood cancers. Transforming genes have not been found in gliomas induced by transplacental carcinogens. Therefore another approach was tried: to introduce transforming genes into the developing nervous system and to characterise the neoplastic response of susceptible cell types. The oncogenes were introduced using replication-deficient retroviral vectors, incubated with a cell suspension from forebrain anlage of foetal rats. The cells, subsequently transplanted into the brain of syngeneic recipients, integrated well and underwent abundant cell proliferation. After several months, spindle cell tumours developed in 40% of the grafts infected with V-*Ha-ras*. Immunohistochemistry revealed expression of S-100 protein but not GFAP, indicating glial origin though they did not resemble oligodendrogliomas. While a viral *gag-myc* construct produced only one tumour, with primative neuroectodermal cells and giant neurons, the combination of viral *ras* and *gag-myc* was extremely effective, rapidly inducing multiple rapidly-growing tumours, highly undifferentiated and negative for all markers including GFAP. After several passages in culture some did express GFAP upon retransplantation, suggesting derivation from glial precursor cells. Both oncogenes were strongly expressed in the tumour cells. The *ras-myc* construct also efficiently transformed neural cells *in vitro*, which upon transplantation produced a neoplasm with architecture typical of primitive neuroectodermal tumour of childhood. Transcripts of v-*Ha-ras* and V-*myc* or *p21ras* protein were not detected in this tumour; insertional mutagenesis of a growth-regulatory gene may have occurred. Thus, retrovirus-mediated gene transfer into brain transplant has demonstrated oncogene complementation in the nervous system and a marked cell-specificity of activated oncogenes for transformation of neural cells.

### *Postnatal influences on prenatally initiated neoplasms*

Positive and negative influence of postnatal factors on the development of prenatally initiated neoplasms must be understood for both risk assessment and for management of exposed populations. B. Diwan (Program Resources, Inc., Frederick, MD) has investigated barbiturates, which promote tumours in several epithelia of adult animals. To test this for prenatally initiated tumours, four barbiturates were administered postnatally, barbital, barbituric acid, amobarbital, or PB. ENU was given transplacentally to B10.A mice, a strain sensitive to intestinal tumour induction. The incidence of these was not influenced by postnatal treatment with any of the barbiturates, but the number of thyroid neoplasms was significantly increased by postnatal PB, and renal tubular tumours increased 10-fold after postnatal barbital. None of the barbiturates increased liver tumour incidence; PB decreased their number. In rats postnatal PB treatment did not influence the most common tumours caused by transplacental MNU, neurogenic and kidney, but did promote thyroid neoplasms and increase the number of preneoplastic liver lesions in female offspring. The possible significance of such phenomena for the human was emphasised, in light of the long period of postnatal life.

Just as prenatally-initiated tumours may be later promoted, it may be possible to suppress their appearance by postnatal treatments. This subject was addressed by V. Alexandrov (Petrov Institute of Oncology, Leningrad, USSR), with a review of extensive screening studies for postnatal inhibitors of transplacental carcinogenesis. The model was transplacental ENU in rats, with nervous system and kidney as the primary targets, followed by exposure throughout postnatal life with a variety of extracts, chemicals and drugs. Thiamine, retinol, α-tocopherol, and selenium, all with chemopreventive actions in some animal models, were without effect. Natural tissue factors, extracted from bovine organs and known to influence immune system function, showed promise: factors from thymus, pineal gland, and brain all decreased tumour incidence and/or multiplicity in many cases. Drugs, including the anti-inflammatory indomethacin, the antidiabetics phenformin and bleoformin, theophylline, diazepam, dilantin and lithium, all suppressed the numbers of one or more tumour type. Inhibition was also observed with DFMO, an inhibitor of ornithine decarboxylase; ε-aminocaproic acid; and fumaric (but not succinic) acid; and some plant-derived chemicals. These survey results open hope for postexposure inhibition of human transplancental carcinogenesis, which may be especially critical for situations where known high exposure defines a high-risk population.

### Animal models for human exposure

### *Diethylstilbestrol*

Interest continues in the mechanism of action and risk implications of the best-established human transplacental carcinogen, diethylstilbestrol (DES). J. McLachlan (National Institute of Environmental Health Sciences, Research Triangle Park, NC) reviewed some current ideas. The neonatal mouse is a useful model, exhibiting stages of vaginal morphogenesis and uterine differentiation comparable to the foetal human, and susceptibility to DES causation of benign adenomatous lesions and vaginal adenocarcinoma. Possible mechanisms include cellular transformation *vs* response to altered hormonal milieu. DES and other oestrogens transform fibroblasts in culture in the absence of any oestrogen-receptor mediated effects, including enhanced cell proliferation. The role of oestrogen receptors was studied with the neonatal mouse model. Uterine tumours were induced by DES given neonatally, but not later, and required later oestrogen stimulation for growth. Neonatal uterine epithelium contains nuclear oestrogen receptors in only 10–20% of cells, and only those cells respond to oestrogen with increased gene expression, but oestrogen causes cells lacking oestrogen receptors to divide. An interaction is postulated between oestrogen and epidermal growth factors (EGF): pro-EGF in the membranes of uterine epithelial cells is processed to free ligand in response to oestrogen and cell division ensues. Thus proliferation may be stimulated in the absence of oestrogen receptor before acquisition of differentiated characters; these may be the target cells for oestrogen-mediated transformation. This idea is being pursued by transfection of the oestrogen receptor gene to fibroblasts in culture, and in transgenic mice overexpressing growth factors in the uterus.

Implications of prenatal DES exposure for mammary tumorigenesis were examined by E. Boylan (Queens College, City University of New York) using the ACI rat. Prenatal DES resulted in a dose-dependent incidence of tumours, and the non-neoplastic mammary tissue presented a wider range of expansion and morphological differentiation than normal, with underdevelopment in some rats and proliferation in others, especially those with tumours. Further experiments attempted to distinguish between direct cellular effects of the DES and those mediated hormonally. After a high transplacental dose of DES and ovariectomy, pellets containing small amounts of DES or oestradiol, for local effect only, were implanted adjacent to the mammary tissue and degree of morphological differentiation scored. The implanted oestrogen stimulated differentiation, but to a lesser degree in those rats that had been exposed to DES prenatally. Thus mammary tissue of such rats was less responsive than controls to the differentiation-enhancing action of directly-applied oestrogen.

C. Lamartiniere (University of Alabama at Birmingham, AL) has investigated the potential of neonatal DES to alter CNS imprinting mechanisms and susceptibility for carcinogenesis. Testosterone from foetal testes, aromatised in the brain to oestrogen, establishes permanent, imprinted sex-specific characteristics, including levels of hepatic enzymes. Oestrogen from foetal ovaries is bound by α-foetoprotein to prevent masculinisation of the brain. DES does not bind to α-foetoprotein and so might alter these imprinting processes, including an eventual effect on levels of carcinogen-metabolising enzymes. This idea was tested in rats, using aflatoxin as a liver carcinogen after neonatal DES and PB in the drinking water as a liver tumour promoter. The incidences of liver neoplasms after aflatoxin-PB exposure were slightly higher in DES-pretreated *vs* control animals. An interesting unexpected finding was a 64% incidence of mammary lesions (mainly lobular hyperplasias and fibroadenomas) in the aflatoxin-PB treated females, but only 5% in those given DES also. Additional studies are required to sort out relative effects of the imprinting dose of DES on spontaneously-arising tumours, on the metabolism of aflatoxin, and on promotive effects of PB.

B. Walker (Michigan State University, East Lansing, MI) considered both DES and maternal dietary fat effects on tumours in offspring of female mice, with models involving prenatal DES; prenatal DES exposure of the mothers; and high dietary fat of mothers with or without their exposure as foetuses to DES. Uterine and ovarian tumours were increasing in offspring in all models, compared with controls. There were also significantly more cervical tumours in those exposed to DES prenatally, increased mammary tumour metastasis after high maternal dietary fat, and more pituitary tumours after DES or high fat. DES might act via direct somatic mutation or by interference with hypothalamic imprinting (see above). Experiments involving blastocyst transplantation and limitation of the period of fat exposure are in progress to reveal critical exposure times and shed light on the mechanism of these effects.

### *NNK*

A. Castonguay (Laval University, Quebec, Canada) summarised data on the transplacental effects of the potent tobacco-specific carcinogen NNK. For DNA damage and tumourigenicity it requires metabolic activation, probably by target tissue. He studied ability of foetal tissues to activate NNK; transplacental passage of NNK; and transplacental carcinogenicity. Lung and tracheal explants from foetal hamsters catalysed α-carbon oxidation of NNK, an activating event, to an extent that increased with foetal age. Explant DNA was methylated at the $0^6$ position of guanine, a promutagenic lesion. Foetal tissue homogenates incubated with NNK and V79 cells caused an increase in the incidence of NNK-caused micronuclei. Tracheal explants incubated with NNK exhibited a dose-related squamous metaplasia. After 500 mg $kg^{-1}$ to pregnant hamsters, both the compound and two metabolites, NNAL and NNK-N-oxide were found in foetal tissues, including binding to lung protein. After intratracheal exposure, to model human smoking more closely, the level of NNK was higher in amniotic fluid than in maternal plasma and was clearly detectable in foetal lung and liver. Evidence of *in vivo* genotoxic effects included micronuclei in foetal liver and dose-related chromatid aberrations in the epithelial cells of lung explants. There was a dose-dependent transplacental carcinogenic effect, to a maximum 70% tumour incidence in the offspring, most often in the respiratory tract. Finally, investigation of interactions of NNK with alcohol indicated an influence on metabolism of NNK in both maternal and foetal tissues.

### *Infiltrating angiolipoma of skeletal muscle in primates*

S. Rehm (National Cancer Institute, Frederick, MD) described infiltrating angiolipoma in 35% of patas monkeys exposed transplacentally to ENU for 12 weeks from gestation day 30. These tumours occurred only after transplacental treatment. The only other species in which they have been described is the human, typically, young adults. The gross and histological features of the tumours are similar in the patas and in humans, with equal sex distribution, occurrence at various locations, and contributions of blood vessels and adipose tissue. While the mitotic rate may be low and metastasis does not occur, they infiltrate skeletal muscle and can become quite large. This may be a useful lead to epidemiological follow-up.

### Epidemiology

Epidemiology studies of perinatal carcinogenesis have focused on human childhood cancer. A. Olshan (University of Pittsburg, PA) provided a summary overview. Since childhood cancer is relatively rare, recent studies have involved case ascertainment from ongoing national collaborative clinical trials conducted by the Children's Cancer Study Group and the Pediatric Oncology Group. A recent case-control study of Wilms' tumour confirmed increased risk for children of fathers employed as vehicle mechanics, auto body repairment, and welders. No association for maternal exposures but a possible link with household insecticides were noted. A case-control study of rhabdomyosarcoma found associations with maternal occupation as health technicians, nurses, and dental hygienists, diagnostic X-ray exposure during pregnancy, and parental use of marijuana or cocaine. In progress are studies of: childhood Hodgkin's disease examining infectious diseases as potential risk factors, including assays of antibody levels in sera samples and of viral genome in histological samples; maternal use of nitrosatable drugs during pregnancy and risk of childhood brain tumours; and the contribution of paternal occupation, drug use during pregnancy, and electromagnetic field exposure on neuroblastoma, including biological markers such as DNA ploidy, cytogenetics, and N-*myc* amplification.

G. Bunin (Children's Hospital, Philadelphia, PA) continued this theme with focus on retinoblastoma, thought to involve two genetic mutations at the Rb locus. There are three circumstances of occurrence: inheritence of a mutated gene from a parent; sporadic heritable retinoblastoma, characterised by bilateral tumour with no family history and implying a new germline mutation; and nonheritable, presumably involving two somatic postconception mutations. A case-control study with 201 pairs (9% with familial, 33% with sporadic heritable, and 57% nonheritable cases) indicated that paternal occupation in metal manufacturing, particularly preconceptionally, was associated with sporadic heritable disease. Paternal preconception military service also correlated but not service in Southeast Asia or exposure to dioxin. An occupational grouping including machinists, welders, and other metal workers was associated with nonheritable retinoblastoma, similarly for pre- and postconception employment. Maternal grandparent's occupations were also examined: a strong association was found between the nonheritable retinoblastomas and grandparental occupation as farmers. Stored body burdens of pesticides in the mothers were suggested. Future plans include integration of molecular biology with epidemiology, for example, examination of specific changes in the retinoblastoma gene as related to paternal exposures.

### Comments

A purpose of this Workshop was to clarify goals of the perinatal carcinogenesis field, and to this end some common themes emerged. (1) The time is past for narrow, self-contained dissection of phenomenology. Animal models should be relating to specific human cancer questions, especially ones that are not easily addressable by application of molecular biology to human material. Consideration of molecular/biochemical factors should ideally be done side-by-side with human and rodent material, and since human material is often unobtainable, nonhuman primates may be particularly valuable in this regard. (2) Epidemiologists on the other hand should be working from thorough, detailed knowledge of animal model results, and more frequent dialogue between epidemiologists and experimentalists is needed. (3) The extent to which perinatal exposures influence incidence of adult-type cancers in humans remains an unplumbed but very important question. Interaction of oncogenic agents with the immature cells of the perinate would be expected to yield the typical undifferentiated tumours of childhood. On the other hand, in rodents, transplacentally-initiated tumours are often morphologically similar to those caused in adults; tumours caused by low, environmental levels of carcinogen might have long latency; and results with DES or high fat exposure during pregnancy, and data on postnatal promoters and inhibitors, suggest persistent changes, some of them subtle, that can continue to be played upon by other influences throughout postnatal life. This question, of origin of typical aging-related human cancers from perinatal exposures, is a challenge for epidemiologists for obvious reasons; additional, carefully planned work with animals could help guide their efforts.